# Dose-dependent effects of calorie restriction on gene expression, metabolism, and tumor progression are partially mediated by insulin-like growth factor-1

**DOI:** 10.1002/cam4.23

**Published:** 2012-08-06

**Authors:** Leticia M Nogueira, Jackie A Lavigne, Gadisetti V R Chandramouli, Huaitian Lui, J Carl Barrett, Stephen D Hursting

**Affiliations:** 1 Division of Cancer Epidemiology and Genetics, National Cancer Institute, NIHBethesda, Maryland; 2 NCI Center for Bioinformatics/Science Applications International CorporationRockville, Maryland; 3 Science Applications International CorporationRockville, Maryland; 4 Translational Sciences for Oncology Innovative Medicines, AstraZeneca Pharmaceuticals, Inc.Waltham, Massachusetts; 5 Department of Nutritional Sciences, University of Texas at Austin and Department of Molecular Carcinogenesis, UT-MD Anderson Cancer CenterSmithville, Texas

**Keywords:** Cancer biology, cancer prevention, carcinogenesis, nutrition

## Abstract

The prevalence of obesity, an established risk and progression factor for breast and many other cancer types, remains very high in the United States and throughout the world. Calorie restriction (CR), a reduced-calorie dietary regimen typically involving a 20–40% reduction in calorie consumption, prevents or reverses obesity, and inhibits mammary and other types of cancer in multiple tumor model systems. Unfortunately, the mechanisms underlying the tumor inhibitory effects of CR are poorly understood, and a better understanding of these mechanisms may lead to new intervention targets and strategies for preventing or controlling cancer. We have previously shown that the anticancer effects of CR are associated with decreased systemic levels of insulin-like growth factor-1 (IGF-1), the primary source of which is liver. We have also reported that CR strongly suppresses tumor development and growth in multiple mammary cancer models. To identify CR-responsive genes and pathways, and to further characterize the role of IGF-1 as a mediator of the anticancer effects of CR, we assessed hepatic and mammary gland gene expression, hormone levels and growth of orthotopically transplanted mammary tumors in control and CR mice with and without exogenous IGF-1. C57BL/6 mice were fed either control AIN-76A diet ad libitum (AL), subjected to 20%, 30%, or 40% CR plus placebo timed-release pellets, or subjected to 30% or 40% CR plus timed-release pellets delivering murine IGF-1 (mIGF-1, 20 *μ*g/day). Compared with AL-fed controls, body weights were decreased 14.3% in the 20% CR group, 18.5% in the 30% CR group, and 38% in the 40% CR group; IGF-1 infusion had no effect on body weight. Hepatic transcriptome analyses indicated that compared with 20% CR, 30% CR significantly modulated more than twice the number of genes and 40% CR more than seven times the number of genes. Many of the genes specific to the 40% CR regimen were hepatic stress-related and/or DNA damage-related genes. Exogenous IGF-1 rescued the hepatic expression of several metabolic genes and pathways affected by CR. Exogenous IGF-1 also rescued the expression of several metabolism- and cancer-related genes affected by CR in the mammary gland. Furthermore, exogenous IGF-1 partially reversed the mammary tumor inhibitory effects of 30% CR. We conclude that several genes and pathways, particularly those associated with macronutrient and steroid hormone metabolism, are associated with the anticancer effects of CR, and that reduced IGF-1 levels can account, at least in part, for many of the effects of CR on gene expression and mammary tumor burden.

## Introduction

Calorie restriction (CR) is a well-established dietary intervention for delaying aging and inhibiting cancer in a variety of animal models [1]. Additionally, restricted calorie diet regimens in various forms (typically in the range of 10–30% of usual energy intake) are the most commonly recommended strategy in humans for preventing or reversing obesity [2], an issue of growing concern in the United States (where >35% of women are obese) and throughout the world [3]. Obesity increases the risk of developing and/or dying from a host of diseases, including breast cancer in postmenopausal women [4]. In the United States, approximately 15–20% of breast cancer deaths are attributed to excess body weight [5], one of the few modifiable risk factors for breast cancer [6]. As breast cancer is the most frequently diagnosed cancer in postmenopausal women in the United States and ranks second as a cause of female cancer death [7], effective intervention strategies for breaking the obesity–postmenopausal breast cancer link are urgently needed

The beneficial effects of CR, typically involving a 20–40% reduction in total energy intake, but isonutrient for vitamins, minerals, fatty acids, and amino acids relative to an ad libitum [AL] fed control diet regimen) on mammary tumor burden in experimental models are well documented [1]. Unfortunately, the mechanisms underlying the anticancer effects of CR are not well established, though evidence has suggested some role for the circulating growth factor, insulin-like growth factor-1 (IGF-1). We and others have shown that exogenous IGF-1 infusion in CR mice ablates many of the anticancer effects of CR [8, 9], suggesting IGF-1-dependent processes play a major role in the CR response. We have also shown that tumor development in liver-specific IGF-1 deficient (LID) mice, which have a ∼75% reduction in systemic IGF-1 levels relative to wild-type mice, is inhibited to levels observed in wild-type mice on a CR regimen [10, 11]. These findings suggest that IGF-1 may be a key mediator of the anticancer effects of CR. IGF-1 exerts its effects on cellular growth and metabolism, at least in part, through activation of the phosphatidylinositol 3-kinase (PI3K)/Akt/mammalian target of rapamycin (mTOR) pathway [12, 13]. We and others have shown that treatment with the mTOR inhibitor rapamycin partially mimics the effects of CR [10, 14, 15]. However, our studies in LID mice and our IGF-1 replacement studies suggest that while IGF-1 is an important mediator of energy balance effects on cancer, other pathways that interact with or are independent of the IGF-1/mTOR pathway are clearly involved as well, and these interactions have not been well characterized.

As CR-related changes in gene expression can markedly affect the physiological state of an organism, gene expression microarray approaches have been useful in identifying previously unknown CR-responsive genes in a tissue-specific fashion [16]. However, these studies have typically used a single CR dose in a single tissue, with a focus on aging-related pathways, and no particular set of genes or pathways common to different organ systems has been identified from these studies to date. We have previously described the effects of CR, across a range of 20–40% CR (and with or without exogenous IGF-1 infusion), on body composition and serum hormones [8]. This study uses tissues from that same study to evaluate (for the first time to our knowledge) the effects of CR across a CR dose range of 20–40%, and with/without IGF-1 infusion, on the hepatic and mammary gene expression. As the liver is a critical metabolic tissue and the main production site of IGF-1 and many other energy balance-related factors, understanding the effect of CR on the hepatic transcriptome and identifying which CR-responsive genes are IGF-1 dependent or independent in liver is important. In addition, as dietary energy balance is one of the few known modifiable factors associated with breast cancer progression, we assessed the effects of CR, with or without exogenous murine IGF-1 infusion, on mammary gland expression of cancer-related genes along with mammary tumor progression and cell signaling in an orthotopic MMTV-Wnt-1 transplant model.

## Materials and Methods

### Animal study design for hepatic microarray experiment

Mice used for hepatic transcriptome analysis were maintained according to the guidelines of the Animal Care and Use Committee of the National Cancer Institute, and the committee approved this study. Forty-five 5-week-old female C57BL/6NCr mice were obtained from the NCI-Frederick Animal Production Area, and, after 1 week, the mice were randomized (*n* = 5 per group) to one of the six groups: (1) control, (2) 20% CR, (3) 30% CR, (4) 30% CR + IGF-1, (5) 40% CR, or (6) 40% CR + IGF-1. All mice were singly housed and received water AL. Control mice received AIN-76A diet (Bio-Serv Corp., Frenchtown, NJ) AL. Mice in the 20% CR group received a daily aliquot of control diet equal to 80% of the mean daily control consumption. CR mice (30% and 40%) received modified versions of the AIN-76A diet formulated such that, when provided as daily aliquots equal to 70% or 60%, respectively, of the mean daily control consumption, the reduction in calorie intake was entirely due to carbohydrates; intakes of all other nutrients were equivalent to those in the control group. All mice received a timed-release pellet (Innovative Research of America, Sarasota, FL) delivering either vehicle or 24 *μ*g/day recombinant murine IGF-1 obtained from PeproTech, Inc. (Rocky Hill, NJ). Body weight and food consumption data were recorded weekly beginning 1 week prior and 4 weeks after randomization to the diet regimens. At the study endpoint (when the mice were 10 weeks of age, a point when the mammary glands are fully mature) all mice were killed under continuous CO_2_/O_2_ exposure, and serum, liver, and mammary fat pads were frozen and stored at −80°C. Necropsied carcasses were also frozen and stored at −80°C for subsequent body composition analyses using dual-energy X-ray absorptiometry according to previously described methods [8]. Serum IGF-1 was measured using a rat IGF-1 radioimmunoassay (RIA) that recognizes both rat and mouse IGF-1 (Diagnostic Systems Laboratories, Inc., Webster, TX). Average values were calculated for two determinations made on a single aliquot of serum from each animal [8].

### RNA isolation

Total liver RNA was isolated using a combined Trizol/Qiagen RNeasy Midi Kit (Qiagen, Valencia, CA) method for hepatic microarray analysis. Tissues were homogenized, centrifuged at low speed, and supernatant was combined with Trizol for RNA extraction as previously described [17].

### Hepatic transcriptome analysis

The Affymetrix M430 A2.0 Array (Affymetrix, Santa Clara, CA) was used for liver gene expression analysis addressing three questions: (1) which hepatic genes are responsive to CR at varying levels of restriction; (2) which CR-responsive genes are dependent on circulating IGF-1 levels; and (3) which CR-responsive genes are IGF-1-independent. Probe synthesis was performed according to the Affymetrix recommended protocol. The purified cDNAs were then used to synthesize biotin-labeled cRNA using the Enzo BioArray HighYield RNA Transcript Labeling Kit (Affymetrix). The biotin-labeled cRNAs were purified and quantified as described in the Affymetrix protocol. After quantification, the cRNAs were fragmented and hybridization to the chips, and detection of hybridized signal intensities were performed according to the protocols provided by the manufacturer.

Sample normalization was carried out using the robust multichip average (RMA) followed by multiple group analysis comparison using analysis of variance (ANOVA). Pairwise comparisons were performed to identify expression fold differences with false-discovery rate (FDR) set at 0.05.

Genes that presented consistent expression changes between the groups were further analyzed, and genes with expression differences equal to or greater than twofold between treatment and control were selected for functional analysis. Gene ontology (GO) analysis and pathway identification of differentially expressed genes was carried out using DAVID [18].

### Mammary insulin signaling polymerase chain reaction (PCR) array

As many of the CR-responsive genes identified in the hepatic transcriptome analysis related to carbohydrate metabolism and insulin-related signaling, Mouse Insulin Signaling Pathway RT2 ProfilerTM PCR Array (SABiosciences, Frederick, MD) analysis was performed using RNA extracted from the fourth mammary fat pads (excised from the same mice as the livers used in the hepatic transcriptome analysis) using the RNeasy Lipid Tissue Mini Kit (Qiagen Inc., Valencia, CA). RNA was reverse transcribed into cDNA using High-Capacity cDNA Reverse Transcription Kit (Applied Biosystems, Carlsbad, CA). The cDNA was mixed with ready-to-use PCR master mix and added to the 96-well plate containing primers for 84 insulin signaling-related genes. Expression was normalized to multiple housekeeping genes. Expression levels were calculated using the comparative cycle threshold method and expressed as mean fold change as previously described [19].

### Quantitative PCR analysis

Using Multiscribe Reverse Transcriptase and TaqMan assays (Applied Biosystems, Branchburg, NJ), quantitative real-time PCR was used to analyze liver and mammary fat pad mRNA levels (*n* = 3). In brief, reverse-transcribed cDNA was independently analyzed in triplicates for Fabp5, Gck, Acot1, Cdkn1, Serpina12, Hnf4*α*, and Scd1 in liver samples and Dusp14, Fbp1, and Irs2 in mammary fat pad samples. Expression levels were calculated using the cycle threshold method, normalized against Act*β*, and expressed relative to values from control mice, which were set at 1.

### Wnt-1 mouse mammary tumor study

Mice were maintained according to the guidelines of the Institutional Animal Care and Use Committee of the University of Texas at Austin. Forty-five 6-week-old ovariectomized female C57BL/6 mice (Charles River Labs, Inc. Frederick, MD) were randomized into three diet groups (*n* = 15 per group), as described above: (1) Wnt-control, (2) Wnt-30% CR, and (3) Wnt-30% CR + IGF-1. Also as described above, the Wnt-30% CR + IGF-1 group consumed the same diet as the CR group and received approximately 24 *μ*g/day of recombinant murine IGF-1 (PeproTech, Rocky Hill, NJ), administered through an Alzet osmotic minipump, model 2006 (DURECT™, Cupertino, CA). All mice were singly housed to facilitate individual dietary intake assessment, and received water AL. Both the control and 30% CR (without IGF) groups also had osmotic minipumps implanted, but received 0.9% saline as a vehicle control. Two weeks after the osmotic minipumps were inserted, mice were injected with ∼50,000 syngeneic MMTV-Wnt1 mammary tumor cells as previously described [20]. Five weeks after injection, serum and tissues were collected. Tumors were weighed, measured, and fixed in 10% formalin or stored at −80°C. Fasting serum leptin and IGF-1 levels were measured using murine LINCOplex® Multiplex Assays (Millipore, Inc., Billerica, MA) analyzed on a BioRad Bioplex 200 analysis system (Biorad, Inc. Hercules, CA).

### Immunohistochemistry analysis of mammary tumors

Formalin-fixed tumor tissues (*n* = 3 per group) were embedded in paraffin and cut into 4-*μ*m-thick sections and processed for either hematoxylin and eosin (H&E) staining or immunohistochemical staining at the Histology Core Laboratory at the U.T. M.D. Anderson Cancer Center – Science Park Research Division (Smithville, TX). Slides were deparaffinized in xylene and rehydrated sequentially in ethanol to water. Antigen retrieval required microwaving slides for 10 min with 10 mmol/L citrate buffer. Endogenous peroxidase activity was quenched with 3% hydrogen peroxide for 10 min. Nonspecific binding was blocked with Biocare blocking reagent (Biocare Medical) for 30 min at RT. Sections were incubated with the following primary antibodies diluted in blocking buffer: Ki-67 (Dako, Carpinteria, CA; 1:200, 4°C overnight); phospho (p)-IGF-1R_Tyr1131_ and IGF-1R (Cell Signaling, Danvers, MA; 1:50, 4°C overnight); p-Akt_Ser473_ (Santa Cruz Biotechnology, Santa Cruz, CA; 1:50, 1 h RT); Akt (Cell Signaling; 1:100; 4°C overnight) p-GSK-3*β*_Ser9_ (Santa Cruz Biotechnology; 1:50, 1 h RT); and CD31. Slides were washed twice in PBS, incubated with horseradish peroxidase (HRP)-labeled *α*-rabbit secondary antibody (Dako) for 30 min at RT at a concentration of 1:200. Diaminobenzidine was used to develop the antibody staining followed with a hematoxylin counterstain. Images were captured using a light microscope equipped with a digital camera (Leica Camera, Inc., Wetzlar, Germany).

### Statistical analysis

Data are presented as mean with standard error of the mean, unless otherwise stated. Affymetrix gene chip data were analyzed as described above. One-way ANOVA followed by Tukey's Honestly Significant differences test was performed to assess differences in serum adipokine levels between groups of mice in the mammary tumor study. Unpaired Student's *t*-test was used to assess differences in mammary gland gene expression. Tumor weight differences were analyzed using the Mann–Whitney test. SPSS software was used for all tests (SPSS Inc., Chicago, IL) and *P* ≤ 0.05 was considered statistically significant.

## Results

### Hepatic transcriptome analysis

**Figure 1:**
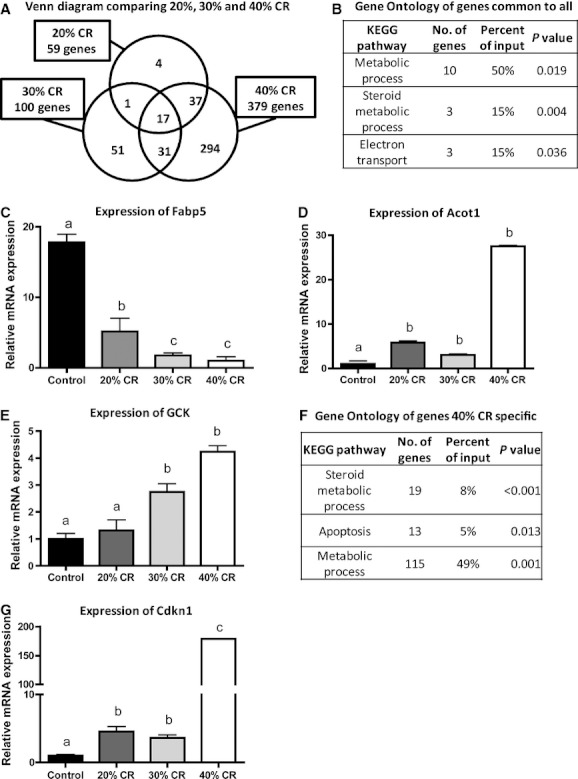
Regulation of gene expression by calorie restriction (CR). (A) Venn diagram comparing 20%, 30%, and 40% CR. (B) Gene ontology of genes common to all. (C) Expression of Fabp5. (D) Expression of Acot1. (E) Expression of Gck. (F) Gene ontology of genes unique to 40% CR. (G) Expression of Cdkn1. Data shown are mean ± SEM, *n* = 5 per group. Significance (*P* < 0.05) between groups is denoted by different letters.

Pairwise gene expression comparisons of hepatic mRNA samples from control versus either 20%, 30%, or 40% CR produced three overlapping groups of 59, 100, and 379 significantly altered genes, respectively, at ≥twofold (Fig. 1A). A total of 14 genes were identified as being common to all CR groups (Table 1).

**Table 1:** Gene expression changes in 20%, 30%, and 40% calorie restriction (CR) compared with control

		Fold change		
		
Gene symbol	Gene title	20% CR	30% CR	40% CR
Hsd3b5	Hydroxy-Δ-5-steroid dehydrogenase	−4.15	−7.37	−13.36
Fabp5*	Fatty acid-binding protein 5	−3.68	−8.34	−25.94
Ugt2b37	UDP glucuronosyltransferase 2 family	−3.21	−3.55	−5.34
Serpina12	Serine (or cysteine) peptidase inhibitor	−2.67	−4.60	−9.75
Cyp51	Cytochrome P450	−2.10	−2.05	−3.07
Gnat1	Guanine nucleotide-binding protein	−2.10	−2.16	−2.96
Xlr3a	X-linked lymphocyte-regulated 3a	−2.07	−2.08	−2.69
Cyp39a1	Cytochrome P450	2.12	2.09	5.12
Fmo3	Flavin-containing monooxygenase 3	2.15	2.49	3.24
Pcp4l1	Purkinje cell protein 4-like 1	2.21	2.88	3.37
Plin5	Perilipin 5	2.79	2.94	8.45
Zbtb16	Zinc finger and BTB domain containing 16	2.92	−2.45	4.00
Acot1*	Acyl-CoA thioesterase 1	3.61	2.55	8.54
Gm3579	Predicted gene 3579	4.99	4.58	9.77

* Genes displayed more than once in the original list (total of 17 hits).

GO analysis showed significant enrichment among the CR-responsive genes for macronutient metabolism processes (10 genes; *P* = 0.019), steroid hormone metabolic processes (three genes; *P* = 0.004), and electron transport (three genes; *P* = 0.036) (Fig. 1B). Our analysis focused on validating and further characterizing the genes related to macronutrient metabolic processes, as these genes are potentially highly relevant to the effects of energy balance in the liver.

Among the 10 genes associated with macronutrient metabolic processes that are common to all CR groups, fatty acid-binding protein 5 (Fabp5) and Acyl-CoA thioesterase 1 (Acot1) were selected for further validation through quantitative real-time PCR. These genes were selected because they participate in lipid metabolism and the peroxisome proliferator-activated receptor (PPAR) signaling pathway, processes important during energy balance changes. Real-time PCR analysis showed that Fabp5 expression decreased in every CR group (4.68 ± 0.83-fold in 20% CR, 10.18 ± 0.13-fold in 30% CR, and 23.60 ± 0.19-fold in 40% CR) compared with control (Fig. 1C). On the other hand, Acot1 was upregulated in all CR groups (5.8 ± 0.9-fold in 20% CR, 3.0 ± 0.5-fold in 30% CR, and 27.5 ± 0.5-fold in 40% CR) compared with control (Fig. 1D).

Only four genes were unique to 20% CR compared to control, with no statistically significant functional category identified. Expression changes for 51 genes were unique to 30% CR compared with control, with significant enrichment of genes related to macronutrient metabolic processes (11 genes; *P* = 0.02). Among these 11 genes, we chose to validate the expression of glucokinase (Gck), as it catalyzes the first rate-limiting step in glycolysis and its transcription is modulated by diet and insulin treatment, as well as by hepatic nuclear factor 4*α* HNF4*α* [21]. When quantitative real-time PCR was run, both 30% CR and 40% CR displayed significantly higher Gck expression (2.7 ± 0.6-fold and 4.2 ± 0.5-fold, respectively) compared with control (Fig. 1E).

In the set of 294 genes specific to 40% CR treatment, GO analysis identified enrichment of genes related to macronutrient metabolic processes (115 genes; *P* = 0.001), genes related to steroid hormone metabolism (19 genes; *P* < 0.001), and genes related to apoptosis (13 genes; *P* = 0.013) (Fig. 1F). Apoptosis is critical for cellular homeostasis, has been associated with the beneficial effects of CR [22], and can be regulated by IGF-1 [23]. Hence, we selected cyclin-dependent kinase inhibitor 1A (Cdkn1a), an important regulator of apoptosis, for further validation. Cdkn1a was expressed 178.7 ± 0.6-fold higher in 40% CR compared with control, but it was also significantly expressed at higher levels in 20% and 30% CR, although with lower expression differences (4.5 ± 1.6-fold and 3.6 ± 0.6-fold, respectively) (Fig. 1G).

**Figure 2:**
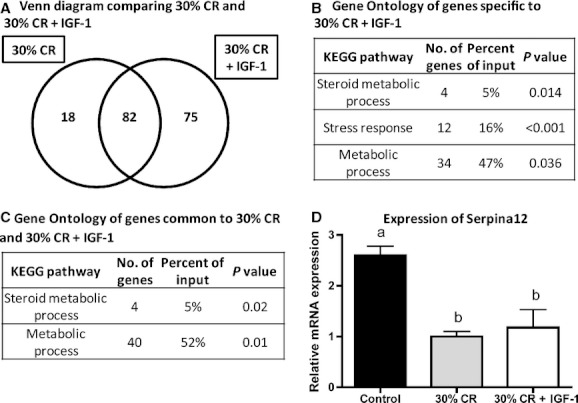
Regulation of gene expression by 30% CR and 30% CR + IGF-1. (A) Venn diagram comparing number of genes expressed by 30% CR and 30% CR + IGF-1 compared to control. (B) Gene ontology of genes specific to 30% CR + IGF-1. (C) Gene ontology of genes common to 30% CR and 30% CR + IGF-1. (D) Expression of Serpina12. Data shown are mean ± SEM. Significance (*P* < 0.05) between groups is denoted by different letters. CR, calorie restriction; IGF-1, insulin-like growth factor-1.

We then compared the list of genes differentially expressed in the 30% CR group with genes differentially expressed in the 30% CR + IGF-1 and control groups to determine which CR-responsive genes are IGF-1 dependent or independent. A total of 18 genes were unique to 30% CR, suggesting that IGF-1 treatment changed the expression of these genes back to levels comparable with control (Fig. 2A), but DAVID analysis revealed no specific categories significantly represented in this set of genes. The 75 genes which displayed significant expression differences between 30% CR + IGF-1 and control, but not 30% CR versus control, contained four genes related to steroid hormone metabolic processes (*P* = 0.014), 12 genes related to stress response (*P* < 0.001), and 34 genes related to macronutrient metabolic processes (*P* = 0.03) (Fig. 2B). This set represents genes for which expression changes were mainly caused by added IGF-1 and not by CR. The 82 genes common between 30% CR and 30% CR + IGF-1 represent genes that were significantly changed by 30% CR, but IGF-1 infusion did not affect expression. Of these, GO identified four genes significantly represented in steroid hormone metabolic processes (*P* = 0.02) and 40 genes related to macronutrient metabolic processes (*P* = 0.01) (Fig. 2C). Using real-time PCR, we validated the expression of Serpin12, an adipokine linked to the metabolic syndrome and insulin sensitivity [24]. It was found that 30% CR decreased the expression of Serpina12 (2.61 ± 0.17; *P* < 0.01), and the addition of IGF-1 did not reverse the effect of CR (2.19 ± 0.35; *P* relative to 30% CR >0.05) (Fig. 2D).

**Figure 3:**
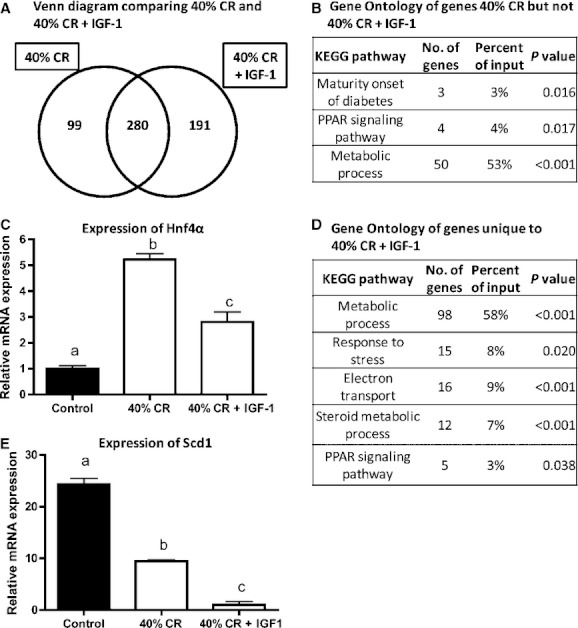
Regulation of gene expression by 40% CR and 40% CR + IGF-1. (A) Venn diagram comparing number of genes differentially expressed for 40% CR and 40% CR + IGF-1, compared with control. (B) Gene ontology of genes uniquely differentially expressed between 40% CR and control, but not 40% CR + IGF-1 and control. (C) Expression of Hnf4*α*. (D) Gene ontology of genes unique to 40% CR + IGF-1. (E) Expression of Scd1. Data shown are mean ± SEM. Significance (*P* < 0.05) between groups is denoted by different letters.

When comparing genes differentially expressed between 40% CR and 40% CR + IGF-1, 280 genes were common between the two, meaning that adding IGF-1 back to 40% CR animals had no effect on the expression of these genes (Fig. 3A). The 99 genes differentially expressed by 40% CR, but not 40% CR + IGF-1, were significantly represented in the maturity onset of diabetes pathway (three genes, *P* = 0.016), the PPAR signaling pathway (four genes, *P* = 0.017), and 50 genes related to macronutrient metabolic processes (*P* < 0.001) (Fig. 3B). This group of 99 genes had their expression restored to levels comparable to control when IGF-1 was infused. We validated the expression of Hnf4*α*, a gene that encodes a transcription factor controlling lipid metabolism in the liver, and is downregulated during hyperinsulinemia [21]. Furthermore, Hnf4*α* activates the transcription of both Gck and glucose-6-phosphatase (G6Pase), depending on fasting or feeding states [21]. Hnf4*α* expression was significantly increased by 40% CR (5.22 ± 0.21-fold). The 40% CR + IGF-1 group showed decreased Hnf4*α* expression compared with 40% CR, although expression level was still significantly higher than control (2.80 ± 0.38-fold) (Fig. 3C), suggesting that IGF-1 partially mediates the effects of CR on this gene.

A total of 191 genes were differentially expressed between 40% CR + IGF-1 and control, but not 40% CR and control, representing expression differences specifically caused by IGF-1 treatment. These genes were significantly overrepresented by macronutrient metabolic processes (98 genes, *P* < 0.001), response to stress (15 genes, *P* = 0.02), electron transport (16 genes, *P* < 0.001), steroid hormone metabolism (12 genes, *P* < 0.001), and the PPAR signaling pathway (5 genes, *P* = 0.03) (Fig. 3D). We further validated the expression of stearoyl-CoA desaturase-1 (SCD1) because it is a critical control point in the development of obesity and insulin resistance. In fact, mice lacking the SCD1 enzyme are lean and protected from diet-induced obesity and glucose intolerance [25]. Relative to controls, Scd1 expression was downregulated in the CR mice (24.33 ± 0.65, *P* = 0.03) and (to a greater extent) the CR + IGF-1 group (2.57 ± 0.30, *P* < 0.001) (Fig. 3E).

### Gene expression changes in the mammary fat pad

**Figure 4:**
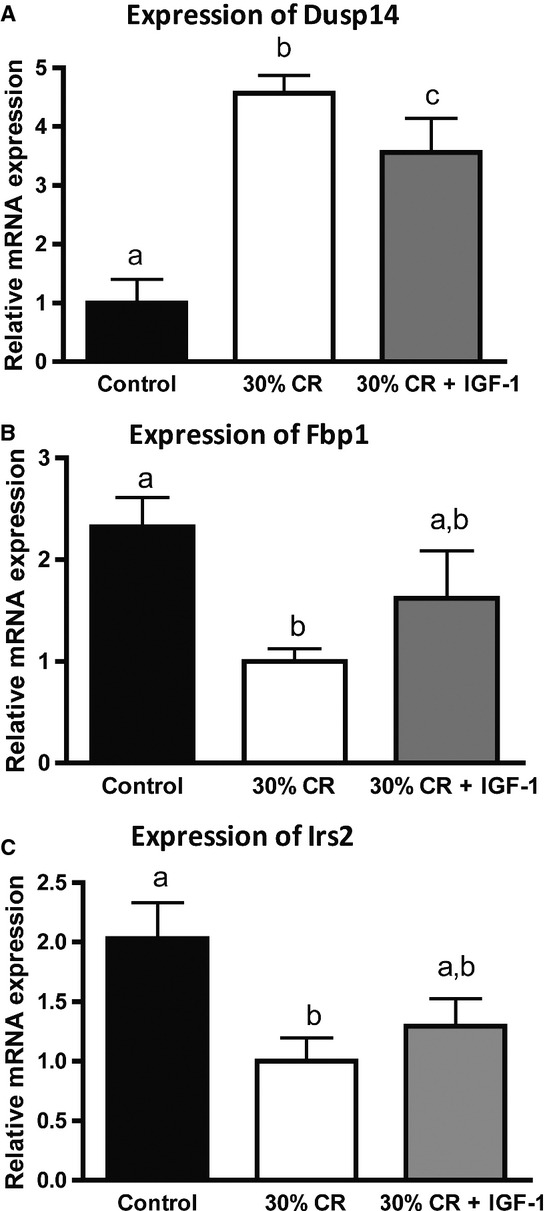
Gene expression changes between 30% CR and 30% CR + IGF-1 in the mammary fat pad. (A) Expression of Dusp14. (B) Expression of Fbp1. (C) Expression of Irs2. Data shown are mean ± SEM. Significance (*P* < 0.05) between groups is denoted by different letters.

To characterize the effects of CR (with and without IGF-1 infusion) in the microenvironment in which mammary tumors arise in mice, we ran Insulin-PCR Super arrays (SABiosciences, Frederick, MD) using mRNA extracted from the mammary fat pads of control, 30% CR, and 30% CR + IGF-1 mice. The Mouse Insulin Signaling Pathway RT^2^ Profiler™ PCR Array profiles the expression of 84 insulin-responsive genes, many of which were found altered in the hepatic transcriptome analysis. We chose to validate the four genes that were differentially expressed between 30% CR and control, but not 30% CR + IGF-1 and control, as these represent genes that were apparently reversed by IGF-1 under CR conditions. Among these genes, Dusp14, a mitogen-activated protein kinase (MAPK) phosphatase, was increased 4.5 ± 0.6-fold compared with control, as validated by real-time PCR (Fig. 4A). This change in DUSP14 expression was diminished in the 30% CR + IGF-1 group compared with 30% CR, though not completely reversed to control levels (3.5 ± 1.2-fold vs. control, *P* = 0.03). This finding suggests the reduction of DUSP14 in response to CR is partially IGF-1-dependent, consistent with the well-established link between MAPK activation and IGF-1 [26]. As fructose biphosphatase 1 (Fbp1) gene expression is controlled by insulin, we also validated its expression by real-time PCR. We found that 30% CR decreased expression of Fbp1 (2.32 ± 0.12-fold), whereas expression was not significantly different from control in the 30% CR + IGF-1 group (Fig. 4B), suggesting that the effect of CR on Fbp1 expression is IGF-1 dependent. Finally, the expression of insulin receptor substrate 2 (Irs2), which is critical for insulin-stimulated glucose uptake [27], was also decreased in 30% CR compared with control (2.02 ± 0.19-fold), but displayed no significant difference between control and the 30% CR + IGF-1 groups (Fig. 4C), suggesting that the effect of CR on Irs2 is IGF-1 independent.

### Wnt-1 mammary tumors

**Figure 5:**
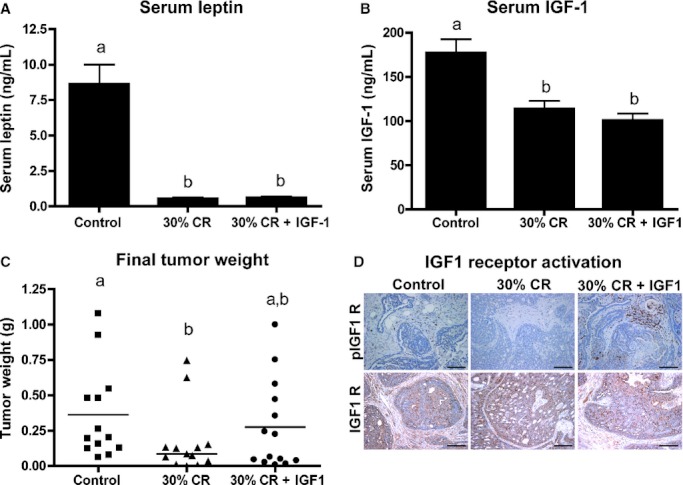
Effects of 30% CR and 30% CR + IGF-1 on mammary tumor growth. (A) Serum leptin levels. (B) Serum total IGF-1 levels. (C) Tumor weight at the end of the study. Data shown are mean ± SEM for serum hormones. Significance (*P* < 0.05) between groups is denoted by different letters. (D) CR tumors showed decreased IGF-1R activation (pIGF-1R) whereas control and 30% CR + IGF-1 showed increased IGF-1R activation. There were no changes in total IGF-1R, pAkt, total Akt, CD31, and Ki67 between the groups. Scale bar = 100 *μ*m.

To elucidate the effects of CR (with and without IGF-1 infusion) on mammary tumor progression, we injected MMTV-Wnt-1 mammary tumor cells in a separate cohort of C57BL/6 mice (*n* = 15 mice/group) that were randomized to receive the same control, 30% CR, and 30% CR + IGF-1 regimens described above. Mice in the 30% CR and 30% CR + IGF-1 groups had significantly lower levels of leptin than controls (Fig. 5A). Leptin has been shown to positively influence breast cancer cell proliferation, invasion, and metastasis in vitro and in vivo [28]. There were no differences in serum insulin and adiponectin levels between the groups (data not shown). Mice in the 30% CR group displayed significantly lower levels of serum IGF-1 (113.7 ± 8.9 ng/mL, *P* < 0.05) compared with control (117.0 ± 15.4 ng/mL), as did mice in the 30% CR + IGF-1 group at this time point (100.8 ± 7.5 ng/mL, *P* < 0.05) (Fig. 5B). As exogenous IGF-1 is cleared very quickly in serum, as previously reported [8], we did not observe an increase in serum IGF-1 levels, but had previously shown that this IGF-1 infusion regimen increases bone density and activation of the IGF-1 receptor in mammary and other epithelial tissues. At the end of the study (5 weeks after injection), tumors in the 30% CR group weighed significantly less than control tumors (*P* = 0.02), consistent with our previous findings of inhibitory effects of CR on mammary tumor growth (Fig. 5C). However, tumor weights from mice receiving the 30% CR + IGF-1 regimen were not statistically different from either CR or control mice, suggesting the IGF-1 infusion partially rescued the reduced mammary tumor growth observed in CR mice.

We then tested whether there were differences between the groups in tumoral IGF-1 receptor (IGF-1R) activation. We measured phosphorylated (p) IGF-1R in tumors by immunohistochemistry. The 30% CR group displayed decreased levels of IGF-1R activation, whereas tumors in the 30% CR + IGF-1 group displayed levels of activation comparable with controls (Fig. 5D). There were no changes in total IGF-1R, indicating that only receptor activation, but not receptor expression, was affected by CR and IGF-1 levels (Fig. 5D). The tumors displayed no changes in cell proliferation, as assessed by staining with Ki67, or angiogenesis, as shown by staining with CD31 (data not shown).

## Discussion

Given the very high and rising prevalence of obesity among U.S. women [7], and the observed obesity/breast cancer associations in epidemiologic studies [29], a better understanding of the factors responsible for the energy balance–breast cancer link is urgently needed to identify new targets and strategies for preventing breast cancer. CR is a well-established, but poorly understood, experimental intervention for preventing/reversing obesity and suppressing the development and progression of mammary and other types of cancers [1]. In this study, we used microarray analysis to identify gene expression changes in response to three CR levels (20%, 30%, or 40% CR relative to AL-fed controls) in the liver, a critical metabolic organ highly responsive to energy balance changes. As the liver is also the primary site for IGF-1 production, and changes in the levels of this growth factor are thought to play a central role in the beneficial effects of CR [1], we also evaluated gene expression changes in the liver of CR mice infused with saline or IGF-1. Finally, we used a MMTV-Wnt-1 syngeneic orthotopic transplant model to evaluate the effects of CR (with and without) infused IGF-1 on mammary tumor growth. Our hepatic gene expression analyses indicated that relative to 20% CR (which was similar to control), 30% CR significantly modulated more than twice the number of genes, and 40% CR more than seven times the number of genes. Many of the genes specific to the 40% CR regimen were hepatic stress-related and/or DNA damage-related genes, suggesting this level of restriction may be too severe. Exogenous IGF-1 rescued the hepatic expression of several metabolic genes and pathways affected by CR, but did not normalize the expression of stress-related genes in the liver, suggesting that the stress-related gene expression changes in response to more severe (i.e., 40%) CR regimens are IGF-1 independent. Exogenous IGF-1 rescued the expression of several metabolism- and cancer-related genes affected by CR in the mammary gland. Furthermore, exogenous IGF-1 partially reversed the mammary tumor inhibitory effects of 30% CR. Thus, CR modulates (in a dose-dependent manner) several metabolic pathways in liver, a key systemic regulator of energy metabolism. CR also alters multiple metabolic- and/or cancer-related genes (many in pathways observed to be CR responsive in liver) in the in mammary tissue, in which tumor development and progression are modulated by dietary energy balance modulation.

Most of the previous CR-related microarray studies in the liver have focused on the effects of CR in aging-related processes [30, 31]. Other studies have evaluated the effects of CR across a time course, showing that changes in gene expression occur fairly early after CR treatment has started [16] and that the effects of CR in aging change depending on the time in life when treatment started [32]. To our understanding, this is the first microarray analysis comparing the effects of variable levels of CR on hepatic and mammary gene expression. We concomitantly analyzed the effects of increasing levels of CR and infused IGF-1 on gene expression in the liver, and identified a list of genes for which expression changes are common between three different levels of CR, as well as a set of genes for which expression is returned to control levels when IGF-1 is infused into CR mice.

First, we hypothesized that increasing levels of CR would lead to increasing qualitative and quantitative effects on gene expression in the liver, which could be evaluated by the number of differentially expressed genes, number of GO categories identified, number of genes in each functional category, and gene expression fold changes. As hypothesized, 30% CR altered the expression of two times more genes than 20% CR, whereas 40% CR altered the expression of approximately seven times more genes than 20% CR. Increasing levels of CR also increased the number of genes in specific GO categories. For example, 40% CR displayed 115 genes associated with metabolic processes, compared with 11 genes in 30% CR. Increasing severity of CR (from 20% to 40% total energy reduction relative to AL-fed controls) also increased differences in expression of several specific genes. Fabp5 was identified as being significantly downregulated in all three CR groups in a dose-dependent manner; 20% CR displayed a fivefold change, whereas 30% CR displayed 11-fold, and 40% CR displayed 25-fold change compared with controls. To our knowledge, this is the first report that CR modulates Fabp5, although its expression has been shown to be increased in human nonalcoholic fatty liver disease [33, 34]. Additionally, apoptosis was identified as a functional category significantly represented in the set of genes unique to 40% CR, which had not been identified in the 20% and 30% CR groups. Higher levels of hepatic apoptosis in CR had been previously reported, although the mechanism responsible for this effect, as well as the consequences, are not well established [22]. The fact that several stress-related genes, as well as Cdkn1, are known to be upregulated following liver damage [35] might mean that 40% CR is detrimental to the liver.

This study design also allowed for the identification of a set of genes for which expression changes are common to different levels of CR. Of the 14 genes identified, 10 were related to macronutrient (particularly lipid and carbohydrate) metabolic processes. Among these, we validated the expression of Fabp5 and Acot1. Fabp5 functions as an antioxidant protein by scavenging reactive lipids in the liver [36, 37] and has been shown to display increased expression in hepatic cells of mice treated with Western Diets [38]. The fact that Fabp5 expression was decreased in every CR group indicates that the levels of scavenging reactive lipids are diminished in the liver during negative energy balance status, compared to the typical high-fat Western Diet. The specific function of Acot1 is not fully understood, but it is thought to increase fatty acid oxidation, decreasing triglyceride formation [39]. It is also thought to be highly regulated at the mRNA level under different physiological conditions by both PPAR and HNF4*α* [40].

We then compared changes in hepatic gene expression between 30% CR and 30% CR + IGF-1, and 40% CR and 40% CR + IGF-1 to identify CR-responsive genes that are IGF-1 dependent and independent. We focused on genes that were differentially expressed between CR and control, but not different between CR + IGF-1 and control. These genes are representative of expression changes that were returned to control levels when IGF-1 was added back. In the 40% CR and 40% CR + IGF-1 comparison, at least two functional categories highly relevant for the effects of obesity on breast cancer risk were identified: maturity onset of diabetes and PPAR signaling pathway. Type 2 (maturity-onset) diabetes is a disease associated with obesity and thought to be responsible for some of its consequences in increasing breast cancer risk and worsening prognosis [41]. The PPAR signaling pathway is highly associated with obesity [42] and has also been shown to be important in regulating tumor growth [43]. We validated the expression of Hnf4*α*, a gene downregulated during hyperinsulinemia that also participates in the PPAR pathway, underscoring the importance of IGF-1 to the metabolic effects of CR.

Given that 40% CR is likely too severe to be practically translated in human studies and might be detrimental to the liver, and that 20% CR had minimal effects on gene expression, we decided to focus on the effects of 30% CR on mammary fat pad gene expression and tumor burden. We have previously reported that 30% CR decreases tumor growth in the MMTV-Wnt-1 transgenic mouse model, and that diet-induced obesity enhances tumor growth [20]. We also previously reported that 30% CR differentially affects the mammary transcriptome, relative to treadmill exercise, and that CR particularly impacts genes involved in carbohydrate and lipid metabolism, including Fabp5 and Acot1 discussed above [44]. Here, we first evaluated gene expression changes in the mammary fat pad between 30% CR and 30% CR + IGF-1 using insulin PCR array. We focused on genes following the same pattern of expression: differentially expressed by 30% CR, but not by 30% CR + IGF-1, compared with control. Among these, Dusp 14 (also found to be CR responsive in [44]) was identified and its expression validated. Dusp14 is a MAPK phosphatase responsible for dephosphorylating ERK, JNK, and p38, and thus, of extreme importance as a downstream signal in the IGF-1 pathway in regulating mammary tumor growth. That Dusp14 inhibits the MAPK pathway and its expression was increased by 30% CR, but not different from control when IGF-1 was added, indicates that IGF-1 is of central importance to the beneficial effects of CR in mammary tumorigenesis. Another gene, namely Fbp1, followed the same pattern of expression between the groups, but in a different direction. Fbp1 was downregulated by 30% CR, but not by 30% CR + IGF-1 in the mammary fat pad. Fbp1 is under the control of insulin and is one of the major regulators of glycolysis [45]. Decreased expression of Fbp1 has recently been associated with tumor growth control [46] consistent with the results obtained in our study. Gene expression changes modulated by energy balance in the mammary fat pad, and the relevance of these gene expression changes to breast cancer, have been previously investigated [44], but this is the first time, to our knowledge, that analyses of the effects across a range of CR levels with and without IGF-1 infusion have been reported.

Finally, we analyzed the effects of 30% CR, with and without IGF-1 infusion, on mammary tumor growth using a syngeneic orthotopic transplant mammary tumor model. We found that 30% CR decreased tumor burden, as measured by final tumor weight, compared with control (*P* = 0.02), whereas 30% CR + IGF-1 displayed no significant difference between either 30% CR or control. This indicates that IGF-1 is a strong component of the CR effects in tumor growth, but is not sufficient for reversing all CR effects. These findings are in contrast to a report of a lack of effect of IGF-1 infusion on the anticancer effects if CR in a chemically induced mammary tumor model in rats, [47]. Possible explanations for the lack of effects of IGF-1 in that study include the short infusion period (8 days) as well as the fact that recombinant human IGF-1 was used. Our findings are consistent with previous reports of the ability of IGF-1 infusion to reverse the anticancer effects of CR in models of mammary, pancreas, bladder, and hematopoietic cancers [10, 23, 48, 49].

Although, as expected, serum IGF-1 levels were not different between 30% CR and 30% CR + IGF-1 at the end of the study, given the rapid clearance of exogenous IGF-1 unbound to IGF-binding proteins, IGF-1 receptor activation in tumors was decreased in 30% CR, but not 30% CR + IGF-1 compared with control. Berrigan et al. previously reported that infused murine IGF-1 leads to increased serum levels of this hormone during the initial period of infusion, with decreased levels toward the end of the study [8]. Here, we show that increasing levels of CR lead to increasing metabolic responses in the liver. While 20% CR did not allow for the identification of any specific gene category, 40% CR appeared to be detrimental to the liver, indicating that 30% CR is an appropriate level of restriction for future mechanistic studies.

There are several limitations to these studies that require consideration. The first is the relatively small number of replicates (five per group) used in the microarray analyses. While more replicates would have been preferred, budgetary constraints prevented increasing the number of gene chips used. Numerous sources suggest five arrays per treatment group can provide adequate replication for such analyses [50, 51]. To increase our confidence in the results, we also repeated the entire array process on a select number of mRNA samples, with nearly identical results (data not shown), and we validated several key targets identified in the array analyses by qRT-PCR, as described above. Another limitation of our studies is the relatively short exposure period (4 weeks) to the diets or IGF-1 infusion. It is possible that a different profile would have been observed if a longer period of CR had been employed, so we can only draw conclusions about short-term CR from our studies. We have shown in other time-course studies that metabolic, body composition, and cellular signaling measurements appear to be quite stable from 4 to 20 weeks of CR; so, given that our focus was on different levels of CR rather than timing of exposure, we thought this was a reasonable strategy. One other limitation is that due to budget constraints, we restricted our studies to a single mouse strain, and a single mammary tumor model. We are planning to extend our gene expression findings to a broader range of models in the future.

In conclusion, several genes and pathways, particularly those associated with macronutrient and steroid hormone metabolism in liver and mammary tissue, are associated with the anticancer effects of CR. Furthermore, reduced IGF-1 levels can account, at least in part, for many of the effects of CR on gene expression and mammary tumor burden. Thus, components of the IGF-1 pathway, as well as components of macronutrient metabolic processes and/or steroid hormone metabolism, represent possible targets for mimicking the anticancer effects of CR.

## References

[1] Hursting SD, Smith SM, Lashinger LM, Harvey AE, Perkins SN (2010). Calories and carcinogenesis: lessons learned from 30 years of calorie restriction research. Carcinogenesis.

[2] World Cancer Research Fund (2007). Food, nutrition, physical activity, and the prevention of cancer: a global perspective.

[3] Flegal KM, Carroll MD, Ogden CL, Curtin LR (2010). Prevalence and trends in obesity among US adults, 1999–2008. J. Am. Med. Assoc.

[4] Calle EE, Kaaks R (2004). Overweight, obesity and cancer: epidemiological evidence and proposed mechanisms. Nat. Rev. Cancer.

[5] Petrelli JM, Calle EE, Rodriguez C, Thun MJ (2002). Body mass index, height, and postmenopausal breast cancer mortality in a prospective cohort of US women. Cancer Causes Control.

[6] Santen RJ, Boyd NF, Chlebowski RT (2007). Critical assessment of new risk factors for breast cancer: considerations for development of an improved risk prediction model. Endocr. Rel. Cancer.

[7] American Cancer Society (2012). Cancer facts & figures, 2011–2012.

[8] Berrigan D, Lavigne JA, Perkins SN, Nagy TR, Barrett JC, Hursting SD (2005). Phenotypic effects of calorie restriction and insulin-like growth factor-1 treatment on body composition and bone mineral density of C57BL/6 mice: implications for cancer prevention. In Vivo.

[9] Hursting SD, Lavigne JA, Berrigan D (2004). Diet-gene interactions in p53-deficient mice: insulin-like growth factor-1 as a mechanistic target. J. Nutr.

[10] Lashinger LM, Malone LM, McArthur MJ (2011). Genetic reduction of insulin-like growth factor-1 mimics the anticancer effects of calorie restriction on cyclooxygenase-2–driven pancreatic neoplasia. Cancer Prev. Res.

[11] Olivo-Marston SE, Malone LM, McArthur MJ (2009). Genetic reduction of circulating insulin-like growth factor-1 inhibits azoxymethane-induced colon tumorigenesis in mice. Mol. Carcinog.

[12] Taniguchi CM, Emanuelli B, Kahn CR (2006). Critical nodes in signalling pathways: insights into insulin action. Nat. Rev. Mol. Cell Biol.

[13] Pollak M (2008). Insulin and insulin-like growth factor signalling in neoplasia. Nat. Rev. Cancer.

[14] Checkley LA, Rho O, Moore T, Hursting S, DiGiovanni J (2011). Rapamycin is a potent inhibitor of skin tumor promotion by 12-O-tetradecanoylphorbol-13-acetate. Cancer Prev. Res.

[15] Harrison DE, Strong R, Sharp ZD (2009). Rapamycin fed late in life extends lifespan in genetically heterogeneous mice. Nature.

[16] Cao SX, Dhahbi JM, Mote PL, Spindler SR (2001). Genomic profiling of short- and long-term caloric restriction effects in the liver of aging mice. Proc. Natl. Acad. Sci. USA.

[17] Takahashi Y, Lavigne JA, Hursting SD (2004). Using DNA microarray analyses to elucidate the effects of genistein in androgen-responsive prostate cancer cells: identification of novel targets. Mol. Carcinog.

[18] Huang da W, Sherman BT, Lempicki RA (2009). Systematic and integrative analysis of large gene lists using DAVID bioinformatics resources. Nat. Protoc.

[19] Narvaez CJ, Matthews D, Broun E, Chan M, Welsh J (2009). Lean phenotype and resistance to diet-induced obesity in vitamin D receptor knockout mice correlates with induction of uncoupling protein-1 in white adipose tissue. Endocrinology.

[20] Núñez NP, Perkins SN, Smith NCP (2008). Obesity accelerates mouse mammary tumor growth in the absence of ovarian hormones. Nutr. Cancer.

[21] Hirota K, Sakamaki J-I, Ishida J (2008). A combination of HNF-4 and Foxo1 is required for reciprocal transcriptional regulation of glucokinase and glucose-6-phosphatase genes in response to fasting and feeding. J. Biol. Chem.

[22] Selman C, Kendaiah S, Gredilla R, Leeuwenburgh C (2003). Increased hepatic apoptosis during short-term caloric restriction is not associated with an enhancement in caspase levels. Exp. Gerontol.

[23] Dunn SE, Kari FW, French J, Leininger JR, Travlos G, Wilson R, Barrett JC (1997). Dietary restriction reduces insulin-like growth factor I levels, which modulates apoptosis, cell proliferation, and tumor progression in p53-deficient mice. Cancer Res.

[24] Wada J (2008). Vaspin: a novel serpin with insulin-sensitizing effects. Expert Opin. Inv. Drugs.

[25] Ntambi JM, Miyazaki M, Stoehr JP (2002). Loss of stearoyl CoA desaturase-1 function protects mice against adiposity. Proc. Natl. Acad. Sci. USA.

[26] Weng CY, Kothary PC, Verkade AJ, Reed DM, Del Monte MA (2009). Map kinase pathway is involved in IGF-1-stimulated proliferation of human retinal pigment epithelial cells (hRPE). Curr. Eye Res.

[27] Fasshauer M, Klein J, Ueki K (2000). Essential role of insulin receptor substrate-2 in insulin stimulation of Glut4 translocation and glucose uptake in brown adipocytes. J. Biol. Chem.

[28] Rose DP, Komninou D, Stephenson GD (2004). Obesity, adipocytokines, and insulin resistance in breast cancer. Obes. Rev.

[29] Morimoto LM, White E, Chen Z (2002). Obesity, body size, and risk of postmenopausal breast cancer: the Women's Health Initiative (United States). Cancer Causes Control.

[30] Bauer M, Hamm Anne C, Bonaus M (2004). Starvation response in mouse liver shows strong correlation with life-span-prolonging processes. Physiol. Genomics.

[31] Miller RA, Chang Y, Galecki AT, Al-Regaiey K, Kopchick JJ, Bartke A (2002). Gene expression patterns in calorically restricted mice: partial overlap with long-lived mutant mice. Mol. Endocrinol.

[32] Dhahbi JM, Kim H-J, Mote PL, Beaver RJ, Spindler SR (2004). Temporal linkage between the phenotypic and genomic responses to caloric restriction. Proc. Natl. Acad. Sci. USA.

[33] Greco D, Kotronen A, Westerbacka J (2008). Gene expression in human NAFLD. Am. J. Physiol. Gastrointest. Liver Physiol.

[34] Westerbacka J, Kolak M, Kiviluoto T (2007). Genes involved in fatty acid partitioning and binding, lipolysis, monocyte/macrophage recruitment, and inflammation are overexpressed in the human fatty liver of insulin-resistant subjects. Diabetes.

[35] Kwon YH, Jovanovic A, Serfas MS, Tyner AL (2002). The Cdk inhibitor p21 is required for necrosis, but it inhibits apoptosis following toxin-induced liver injury. J. Biol. Chem.

[36] Bennaars-Eiden A, Higgins L, Hertzel AV, Kapphahn RJ, Ferrington DA, Bernlohr DA (2002). Covalent modification of epithelial fatty acid-binding protein by 4-hydroxynonenal in vitro and in vivo. J. Biol. Chem.

[37] Zimmer JSD, Voelker DR, Bernlohr DA, Murphy RC (2004). Stabilization of leukotriene A4 by epithelial fatty acid-binding protein in the rat basophilic leukemia cell. J. Biol. Chem.

[38] Hoekstra M, Stitzinger M, van Wanrooij EJA (2006). Microarray analysis indicates an important role for FABP5 and putative novel FABPs on a Western-type diet. J. Lipid Res.

[39] Hunt MC, Alexson SE (2002). The role Acyl-CoA thioesterases play in mediating intracellular lipid metabolism. Prog. Lipid Res.

[40] Dongol B, Shah Y, Kim I, Gonzalez FJ, Hunt MC (2007). The acyl-CoA thioesterase I is regulated by PPAR{alpha} and HNF4{alpha} via a distal response element in the promoter. J. Lipid Res.

[41] Goodwin PJ, Ennis M, Pritchard KI (2002). Fasting insulin and outcome in early-stage breast cancer: results of a prospective cohort study. J. Clin. Oncol.

[42] Barish GD, Narkar VA, Evans RM (2006). PPARI delta: a dagger in the heart of the metabolic syndrome. J. Clin. Investig.

[43] Sporn MB, Suh N, Mangelsdorf DJ (2001). Prospects for prevention and treatment of cancer with selective PPAR[gamma] modulators (SPARMs). Trend Mol. Med.

[44] Padovani M, Lavigne JA, Chandramouli GVR (2009). Distinct effects of calorie restriction and exercise on mammary gland gene expression in C57BL/6 mice. Cancer Prev. Res.

[45] Marín-Hernández A, Rodríguez-Enríquez S, Vital-González PA (2006). Determining and understanding the control of glycolysis in fast-growth tumor cells. FEBS J.

[46] Liu X, Wang X, Zhang J (2009). Warburg effect revisited: an epigenetic link between glycolysis and gastric carcinogenesis. Oncogene.

[47] Zongjian Z, Weiqin J, John M, Pamela W, Thompson H (2005). Effects of dietary energy repletion and IGF-1 infusion on the inhibition of mammary carcinogenesis by dietary energy restriction. Mol. Carcinog.

[48] Hursting SD, Switzer BR, French JE, Kari FW (1993). The growth hormone: insulin-like growth factor 1 axis is a mediator of diet restriction-induced inhibition of mononuclear cell leukemia in Fischer rats. Cancer Res.

[49] Wu Y, Brod YP, Mejia W (2010). Insulin-like growth factor-1 regulates the liver microenvironment and promotes liver metastasis. Cancer Res.

[50] Kang MCK, Yang JJ, McIndoe RA, She JX (2003). Microarray experimental design: power and sample size considerations. Physiol. Genomics.

[51] Pan W, Lin J, Le CT (2002). How many replicates are required to detect gene expression changes in microarray experiments? A mixture model approach. Genome Biol.

