# Correction to “Dose‐dependent effects of calorie restriction on gene expression, metabolism, and tumor progression are partially mediated by insulin‐like growth factor‐1”

**DOI:** 10.1002/cam4.7143

**Published:** 2024-04-01

Leticia M. Nogueira, Jackie A. Lavigne, Gadisetti V. R. Chandramouli, Huaitian Lui, J. Carl Barrett, Stephen D. Hursting. Dose‐dependent effects of calorie restriction on gene expression, metabolism, and tumor progression are partially mediated by insulin‐like growth factor‐1. *Cancer Med*. 2012; 1:275–288. doi: 10.1002/cam4.23

The typo in the co‐author name has been corrected. The corrected co‐author name which is shown below has been added to the online version of the article.

Huaitian Liu.

